# Effects of Induced Structural Modification on Properties of V^+^ Ion-Implanted RF—Magnetron Sputtering Deposited ZnO Thin Films of Thickness 120 nm on Borosilicate Glass Substrates

**DOI:** 10.3390/nano15040278

**Published:** 2025-02-12

**Authors:** Olakunle Oluwaleye, Bonex Wakufwa Mwakikunga, Joseph K. O. Asante

**Affiliations:** 1 Department of Physics, Tshwane University of Technology, P/Bag X680, Pretoria 0001, South Africa; bmwakikunga@csir.co.za (B.W.M.); asantejko@tut.ac.za (J.K.O.A.); 2 Centre for Nano-Structured and Advanced Materials, CSIR, P.O. Box 395, Pretoria 0001, South Africa

**Keywords:** ZnO thin films, structural and optical properties, ion implantation, optoelectronics

## Abstract

The influence of structural modifications on the thermal stability, chemical bonds, and optical properties of zinc oxide (ZnO) thin films (120 nm thick) for optoelectronic devices (solar cells, LEDs) and energy nanodevices was investigated. The films, synthesized via rf-magnetron sputtering, were implanted with V^+^ ions at 170 keV with varying fluences. Optical properties, including bandgap, transmittance, and absorbance, were analyzed using UV–Vis spectroscopy, XRD, AFM, and FTIR. Structural changes such as strain, lattice constant, surface roughness, and crystallite size significantly influenced the optical properties. Increased surface roughness led to a higher optical bandgap (up to 4.10 eV) and transmittance (82.34%), with reduced absorbance (0.12 nm). Crystallite size exhibited similar effects. At an ion fluence of 1 × 10^16^ ions/cm^2^, the bandgap and transmittance increased, while absorbance slightly decreased. Thermal stability and chemical bond analysis supported these findings. The study demonstrates that V^+^ ion-induced modifications enhance ZnO thin films’ properties, highlighting their potential for advanced optoelectronic and energy nanodevice applications.

## 1. Introduction

The fundamental physical principles governing material behavior are well understood through materials physics [1]. However, the behavior of transparent conducting oxides, such as zinc oxide (ZnO) nanomaterials, under ion implantation remains an area requiring further investigation. This requires a detailed understanding of ion–matter interactions at the nanoscale. These interactions produce various non-equilibrium and non-linear states, offering opportunities for developing new materials [1]. Afzal et al. (2024) [2] demonstrated that ion implantation by annealing can effectively modify the electronic properties of transparent conducting oxide (TCO) thin films.

ZnO is a direct band gap semiconductor with a high exciton binding energy at room temperature [3], making it colorless and transparent. This material is essential in optoelectronic and energy nanodevices [3]. Among 3D transition metals (TM), vanadium (V) has a similar ionic radius to zinc, allowing it to incorporate seamlessly into the ZnO lattice structure [4]. Vanadium ions, with their electron configuration [Ar] 3d^3^4s^2^, can substitute Zn^2^^+^ ions, leading to ZnO thin films with unique properties.

Both doping during growth and post-growth ion implantation affect thin film properties based on ion mass, implantation energy, and fluence [5,6]. Various techniques, including RF magnetron sputtering [7,8,9], atomic layer deposition [2], and pulsed laser ablation [10], have been explored for ZnO thin-film modification. For example, research from 2023 explored the effect of copper (Cu) doping on ZnO thin films, particularly concerning electron–hole pair recombination [7]. Another recent study, published in 2024, investigated niobium (Nb)-doped ZnO thin films, focusing on donor defect-induced ferromagnetism [6]. These published studies highlight the versatility of RF magnetron sputtering in fabricating metal-doped ZnO thin films with tailored properties

While extensive studies exist on transition metal-doped ZnO during growth and the improvement of physical properties of optoelectronic and nanodevices [9,11], investigations of structural changes induced by V^+^ ion implantation remain limited. Prior studies [6,12,13,14,15,16] report mixed findings regarding magnetic properties, with some observing ferromagnetism and others not. Further, Han et al. (2024) [9] report on the deposition of zinc-doped indium oxide (IZO) thin films on silicon dioxide substrates using radio-frequency magnetron sputtering, and they realized enhanced transparency and mobility. Toma et al. (2023) [8] suggest that co-doping with Nd and Ga using rf magnetron sputtering can optimize the electrical and optical properties of ZnO films.

The novelty of V^+^ ions on ZnO thin films lies in their unique ability to simultaneously and controllably influence structural, optical, chemical bond, and thermal properties. During implantation, V^+^ ions enable: (a) defect engineering, such as modifying oxygen vacancies (OV) and zinc interstitials, introducing localized states that alter carrier concentration and mobility; (b) improved crystallinity by modifying lattice strain and structural defects, enhancing carrier transport and optical clarity; and (c) impact on surface states, crucial for advanced applications. These effects make V^+^ ion implantation ideal for nanodevices and multifunctional applications. This study focuses on the effects of V^+^ ion-induced structural modifications on optical properties, thermal stability, and chemical bonding in V^+^ ion-implanted ZnO thin films on borosilicate glass substrates.

## 2. Methodology

This study employed two approaches: (i) simulation of ion implantation parameters using Stopping and Range of Ions in Matter (SRIM-2013) and Transport and Range of Ions in Matter (TRIM-2013) [17], and (ii) experimental techniques, including (a) the deposition of ZnO thin films on borosilicate substrates, (b) ion implantation, and (c) characterization of the ZnO thin films.

### 2.1. Simulation

**Figure 1:**
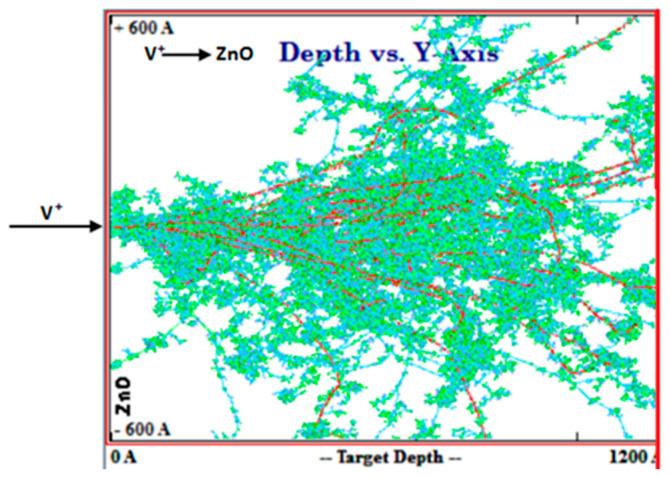
V^+^ ion-implanted ZnO collision cascade within the ZnO thin films and modes of energy loss.

Simulations were crucial to determine the V^+^ ion implantation parameters and estimate the expected experimental results. Theoretical calculations were performed using SRIM-2013 software to simulate V^+^ ion energy, fluence, and ion range. The interactions between V^+^ ions and ZnO thin films (Figure 1) were simulated to analyze dopant concentration, critical energy, total atom displacement, vacancy density, and replacement collisions. These parameters were recorded for each implanted sample.

### 2.2. Experimental Techniques

This subsection describes the synthesis, implantation, and characterization of ZnO thin films on borosilicate glass substrates.

#### 2.2.1. Synthesis of ZnO Thin Films

ZnO thin films were deposited on borosilicate glass substrates using RF magnetron sputtering at the University of the Witwatersrand, Johannesburg, South Africa. The borosilicate glass was cut into 1 cm^2^ sections using a diamond cutter and sequentially cleaned with acetone, ethanol, and ultrapure water in an ultrasonic bath for 10 min each. The cleaned substrates were dried with high-purity nitrogen gas.

The sputtering chamber was evacuated using a turbomolecular pump to a base pressure of ~8 × 10^−6^ Torr before film deposition. Argon gas was used as the ambient gas, with a working pressure of 2 m Torr. ZnO thin films were deposited at room temperature, using a sputtering power of 250 W, for 20–40 min. A 99.99% pure ZnO was used as the target, placed approximately 8 cm from the substrate. To eliminate contaminants, the target was pre-sputtered for 20 min before deposition. Thin films of ~120 nm thickness were deposited on the borosilicate substrates.

#### 2.2.2. Implantation of ZnO Thin Films

The ZnO thin films were annealed at 380 °C for 420 s before implantation. V^+^ ions were implanted at 170 keV using the 200 keV ion implanter at NRF-iThemba LABS, Gauteng, South Africa. V^+^ ions were generated from an ion source using the gaseous form of their elements. These ions were accelerated and directed using electrical and magnetic forces. An analyzer magnet directed the ion beams through variable slits for beam control.

The ZnO thin films were placed in the target chamber for implantation. The ion currents were on the microampere scale, and ion fluence was determined as the integral of the current over time. The penetration range of the ions, fluence, and implantation energy was predetermined through simulation (Table 1). This precise process ensured the controlled modification of the thin film properties.

#### 2.2.3. Characterization of Samples

The thermal stability of the samples, including Debye–Waller factors (B) and mean square vibrational amplitude (U), was estimated using the Wilson plot method with EXPO2014 software [18].

The morphological changes in V^+^ ion-implanted ZnO thin films were studied using an atomic force microscope (AFM) in contact mode, providing topographic properties with resolutions ranging from 100 µm to less than 1 µm. The structural properties of the films were analyzed using X-ray diffraction (XRD) with a PW3373/00CULFFDk184487 diffractometer (Company: Malvern Panalytical, is headquartered in Almelo, Netherlands.. Local office: Malvern Panalytical South Africa (Pty) Ltd., Johannesburg, Gauteng, South Africa. Software: Quantify, an XRD analysis software), employing Cu Kα radiation (λ = 1.54184 Å, 45 kV, 40 mA). Crystallite size and microstrains were estimated using the Williamson–Hall method [19] and GSAS-II crystallography software version 5802 [20].

The Optical properties of the ion-implanted films were studied using a Lambda 750S UV–Vis spectrometer, manufactured by PerkinElmer, Waltham, MA, USA. The Lambda 750S spectrophotometer utilizes the UV WinLab software for instrument control and data analysis. Absorbance (A) and transmittance (T) values were analyzed to determine the optical band gap using Tauc’s relation [21].

## 3. Results and Discussion

### 3.1. SRIM and TRIM 2013 Simulation

Considering the ZnO thin film thickness (Table 1, Column 3), the V^+^ ion energy was chosen to achieve a calculated projected range (Rp), recorded in Column 4 of Table 1.

To analyze the changes induced in ZnO thin films by V^+^ ion implantation, it is essential to understand how V^+^ ions lose energy during their interaction with ZnO thin films. Energy loss occurs in two ways:

Nuclear energy loss: At lower energies, elastic collisions with the nuclei of ZnO lead to energy loss. The incident V^+^ ions transfer energy to ZnO nuclei through these collisions.

Electronic energy loss: At higher energies, inelastic collisions result in the excitation of electrons. This is termed electronic energy loss.

The interaction of V^+^ ions with compound targets such as ZnO thin films is described by Bragg’s rule [22], which assumes statistical independence between the interactions of ions and target atoms, unaffected by atomic binding. The total stopping power (S) of ions penetrating the target and the multiple scattering (Ψ are expressed in Equations (1) and (2), respectively [22]:

(1) $$S=-\frac{dE}{dz}=\sum [N_{i}({\hat{S}}_{n,i}+{\hat{S}}_{e,i})]$$

(2) $$\Psi^{2}=∆z\;\sum [N_{i}(Q_{n,i}+{SQ}_{e,i})].$$

Here, $i\;Q_{n,i}$ and $Q_{e,i}$ represent nuclear and electronic contributions to the multiple scattering cross-section, N is the atomic density, and i refers to the different target elements. Material modification due to nuclear energy loss depends on factors such as defect formation, phase transitions, and the diffusion properties of implanted species [1]. On the other hand, electronic energy loss influences material modification by transferring energy from target electrons to the lattice [1].

Incorporating Equations (1) and (2) into TRIM version 2013 software, the calculated Rp of V^+^ ions in ZnO thin films for the given implantation energy (Table 1, Column 2) is 73.98 nm. This range is less than the ZnO thin film thickness (Table 1, Column 3), ensuring that most V^+^ ions are implanted within the film, where defect-induced modifications occur.

**Figure 2:**
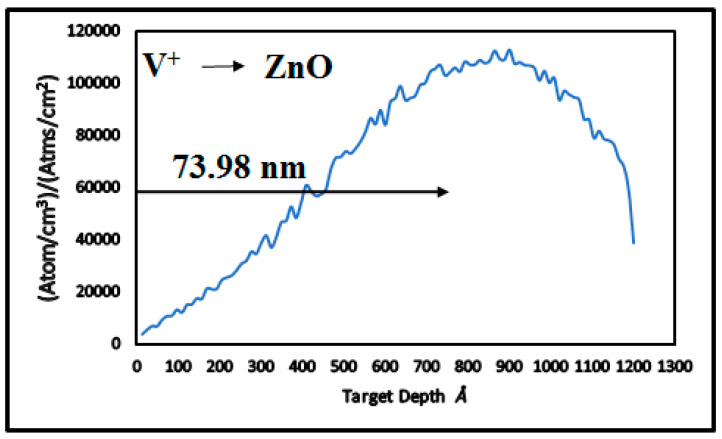
Ion distributions in V^+^ ion-implanted ZnO at 170 keV.

The two-dimensional ion distributions for V^+^ ion-implanted ZnO thin films on borosilicate glass substrates are shown in Figure 2. A simulation of 99,999 ions produced the observed distributions.

**Figure 3:**
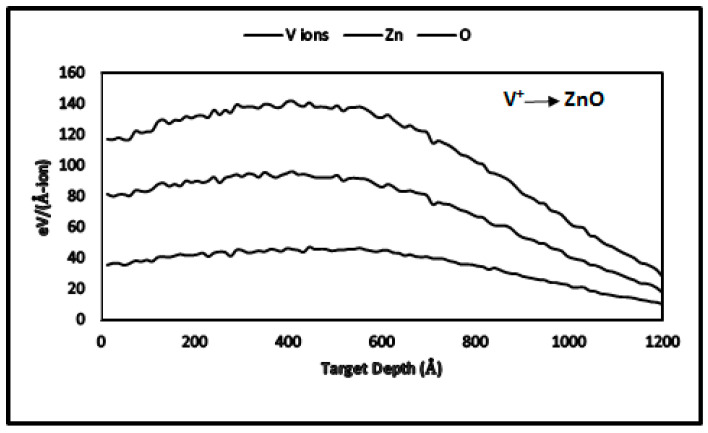
TRIM-2013 plot for energy transferred to recoil atoms in V^+^ ion-implanted ZnO at 170 keV.

The Rp represents the average depth of implanted ions, as calculated in TRIM-2013 (Figure 2, Table 1, Column 4). The dopant concentrations (Table 2, Column 2) were derived from the product of ion fluence and the peak value of the ion distribution plot. Critical energy densities (Table 2, Column 4) were determined by multiplying the fluence by the maximum peak energy required to displace atoms, as shown in Figure 3.

**Figure 4:**
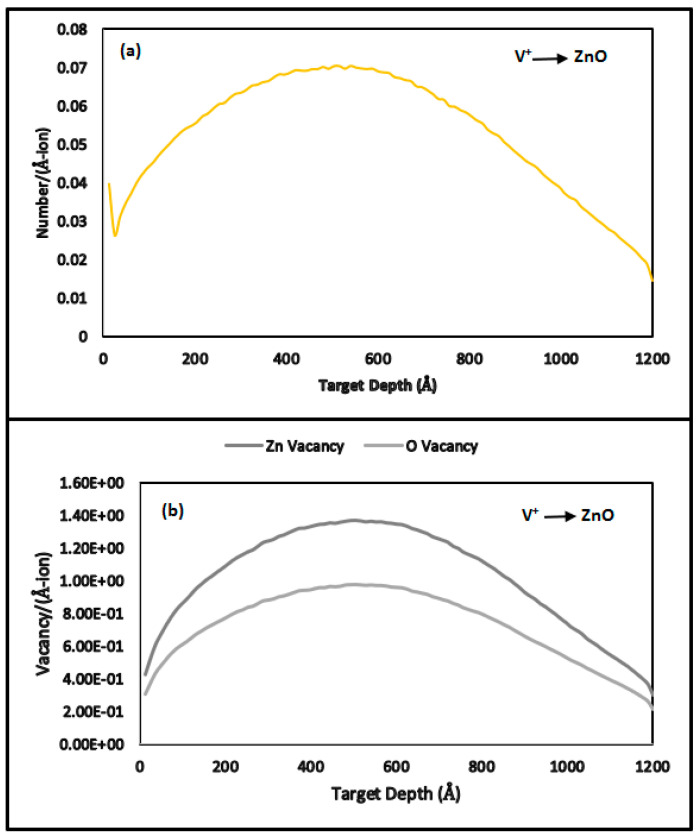
TRIM-2013 plots for V^+^ ion-implanted ZnO at 170 keV. (**a**) Collision events; (**b**) total displacements, vacancies, and replacement collisions.

Figure 4a depicts the collision events of V^+^ ion-implanted ZnO on borosilicate glass substrates, while Figure 4b illustrates the resulting vacancies, replacement collisions, and interstitial atom formations. The maximum vacancy production was 1.36 vacancies per V^+^ ion. Vacancy densities (Table 2, Column 3) were calculated by multiplying 1.36 by the fluence, yielding a displacement energy of 3.92%.

**Figure 5:**
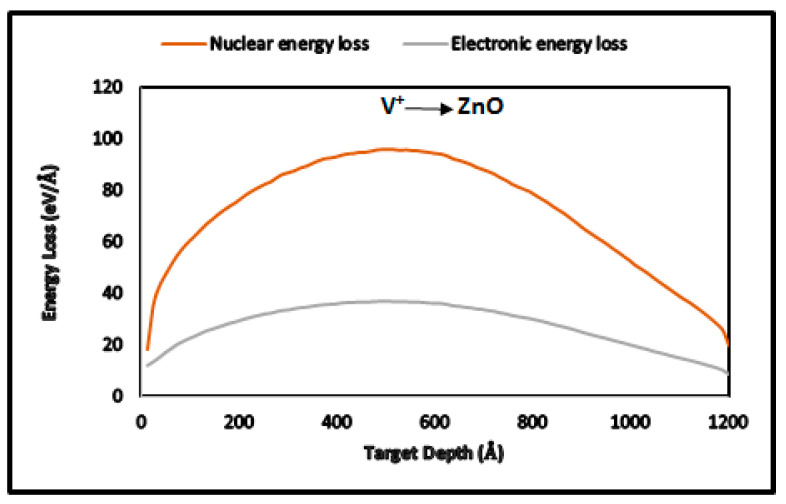
TRIM-2013 plots for nuclear and electronic energy losses in V^+^ ion-implanted ZnO at 170 keV.

As V^+^ ions travel through ZnO thin films, they follow random paths and lose energy through electronic and nuclear collisions. Table 3 and Figure 5 show that electronic energy loss accounts for 41.52%, while nuclear energy loss accounts for 54.27% of the total energy loss. Hence, nuclear energy loss is the dominant mechanism affecting thin-film modification.

**Figure 6:**
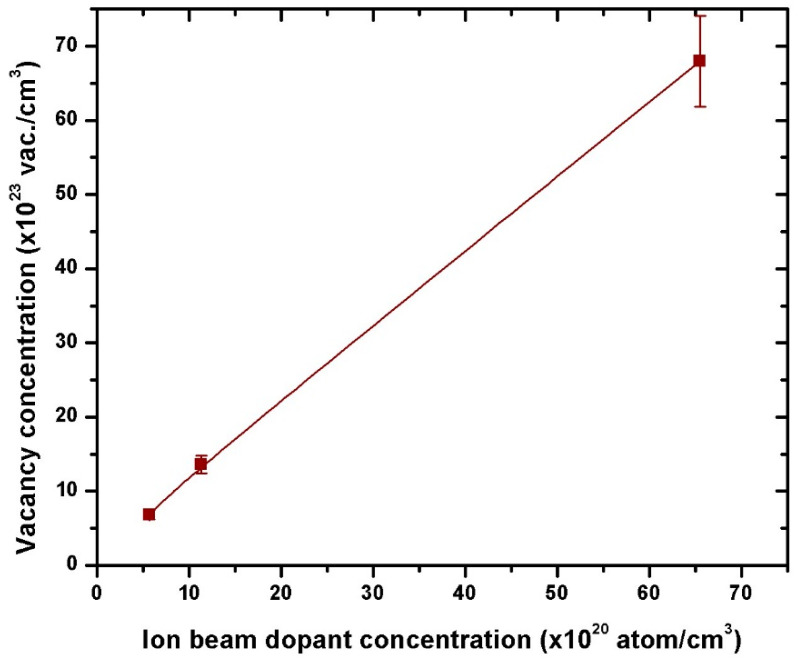
Calculated vacancy concentration as a function of V^+^ ion beam dopant concentration in V^+^ ion-implanted ZnO on a borosilicate glass substrate at 170 keV.

To quantify the extent of modification induced by V^+^ ions, the displacement per atom (dpa) was calculated. With a ZnO density of 8.301 × 10^22^ atoms/cm^3^, dpa values (Table 2, Column 5) were obtained by dividing vacancy density by material density. Figure 6 reveals a linear increase in vacancy concentration with increasing dopant concentrations, from 1.36 × 10^24^ to 6.83 × 10^24^ vac./cm^3^. This behavior is attributed to the increased implantation fluence.

### 3.2. Crystallographic Analysis

#### 3.2.1. As-Grown and V^+^ Ion-Implanted ZnO Thin Films

**Figure 7:**
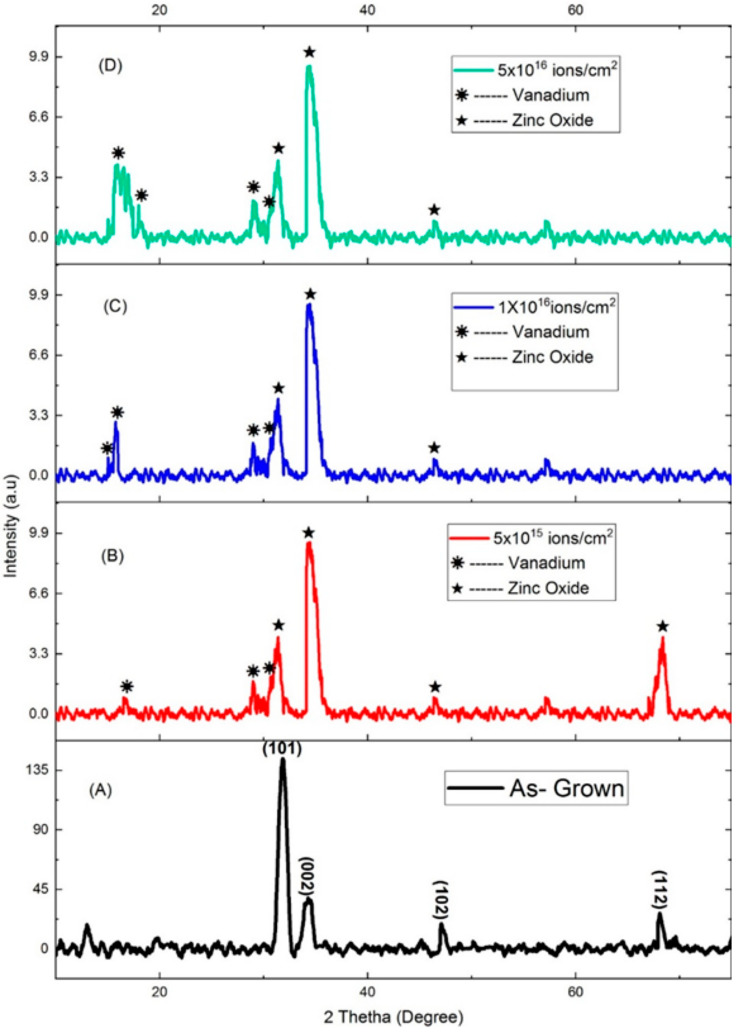
XRD spectra of (**A**) as-grown and V^+^ ion-implanted ZnO thin films at fluences of (**B**) 5 × 10^15^, (**C**) 1 × 10^16^, and (**D**) 5 × 10^16^ ions/cm^2^.

The lattice crystal structures of as-grown and V^+^ ion-implanted ZnO thin films on borosilicate glass substrates were analyzed. Figure 7A–D display the XRD patterns for as-grown ZnO thin films and those implanted with V^+^ ions at various fluences (ranging from 5 × 10^15^ to 5 × 10^16^ ions/cm^2^).

From Figure 7A, the as-grown ZnO thin film exhibits four peaks at 2θ = 31.83, 34.27°, 47.08°, and 68.09°, corresponding to the (101), (002), (102), and (112) planes, respectively. These peaks are attributed to the diffraction of ZnO [23,24]. The most intense (101) peak at 2θ = 31.83° indicates a preferred orientation of the ZnO crystallites with their (101) crystal plane parallel to the borosilicate glass substrate. This preferred orientation is attributed to the thin film thickness of 120 nm.

The V^+^ ion-implanted ZnO thin films (Figure 7B–D) show additional peaks at 2θ = 15.03°, 16.54°, 17.98°, 28.98°, and 30.69°, corresponding to the diffraction from the (200), (001), (101), (110), and (301) planes of vanadium, respectively [25]. These new phases suggest the possible formation of compounds such as vanadium oxide or vanadium-implanted ZnO.

It is noteworthy that the (101) peak at 2θ = 31.83° slightly shifted to 2θ = 31.37°, while the (002) peak at 2θ = 34.27° shifted by 0.18°. With intensity ratio recorded in Table 4 (Column 2), the XRD patterns (Figure 7, Table 5, Column 2) for ion fluences ranging from 5 × 10^15^ to 5 × 10^16^ ions/cm^2^ exhibit a new peak at 2θ = 34.45°, indicating a change in the preferred orientation from the (101) to the (002) crystal plane, alongside a modification in the lattice structure.

As observed in the XRD spectra of as-grown and V^+^ ion-implanted ZnO thin films (Figure 7), the presence of sharp multiphases confirms the polycrystalline nature of the samples. The intensities (I) of the diffraction peaks for V^+^ ion-implanted ZnO thin films change with ion fluence, as shown by the variation in the intensity ratio 100(I/I_max_) recorded in Table 4, Column 2.

**Figure 8:**
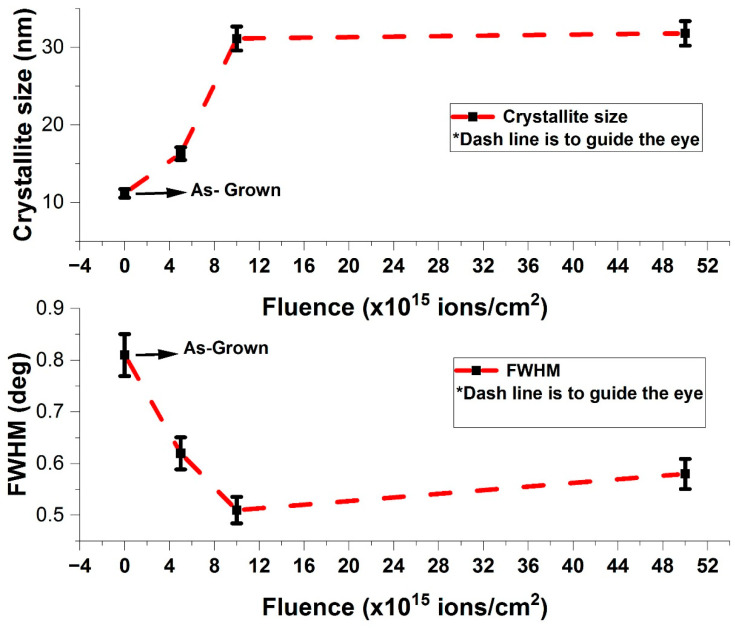
The grain size and FWHM of V^+^ ion-implanted ZnO thin films in comparison with as-grown and in relation with Equation (3) as a function of ion fluence.

From Table 4, Column 2, the intensity ratio changes by 50.04% after ion implantation. This is due to an increase in the number of diffraction peaks, from four in the as-grown ZnO thin films to eight at higher ion fluences (Figure 7). Additionally, the increased crystallite size (Figure 8) suggests that the implantation process reduced the number of defects in the ZnO crystals. As ion fluence increases, the structure undergoes re-crystallization. This observation aligns well with the simulation results discussed earlier.

Therefore, the degree of crystallinity in V^+^ ion-implanted ZnO thin films is higher than that of the as-grown samples. The implantation process improved the crystallinity of the ZnO thin films by reducing defects and enhancing the structural order.

Structural changes observed in XRD (lattice strain, increased crystallite size, defect formation, phase evolution) are driven by the energetic implantation of vanadium ions. XRD evidence shows shifts in the peak positions (2θ), the broadening of peaks, the disappearance of certain peaks, and new peaks corresponding to vanadium oxide phases in the XRD. These structural changes correlate strongly with optical changes (bandgap shifts, transmittance), together with thermal and chemical bonds. These are discussed in detail in the subsequent sections.

#### 3.2.2. Crystallite Size and Strain

The crystallite size of as-grown and V^+^ ion-implanted ZnO thin films was estimated by analyzing the broadening of the observed XRD peaks. The broadening, expressed as the full width at half-maximum (FWHM), is inversely proportional to the crystallite size. Table 5, Column 4, shows the broadening of as-grown and V^+^ ion-implanted ZnO films. The crystallite size, D was calculated using the Debye–Scherrer formula [26,27]

(3) $$D=\frac{0.9\lambda }{\beta cos\theta }.$$

Here, λ represents the X-ray wavelength (1.54060 Å), β is the FWHM in radians, and θ is the Bragg angle.

Equation (3) shows that smaller crystallite sizes result in larger FWHM values. The calculated FWHM and corresponding grain sizes are illustrated in Figure 8 and recorded in Table 5, Column 4, and Table 6, Column 3, respectively. The average FWHM values for as-grown and V^+^ ion-implanted ZnO films at fluences of 5 × 10^15^, 1 × 10^16^ and 5 × 10^16^ ions/cm^2^ were 0.81, 0.62, 0.51, and 0.58, respectively.

The effects of ion fluence on FWHM are evident; as ion fluence increases from 5 × 10^15^ to 5 × 10^16^ ions/cm^2^, the FWHM progressively decreases, leading to an increase in grain size (Figure 8). The grain size increases from 11.18 nm to 31.82 nm, representing a 64.54% increase with ion fluence. This trend aligns with Equation (3) and indicates that ion implantation reduces defects in the ZnO crystals, leading to a decrease in peak broadening and an increase in grain size. The overall crystallinity of the ZnO thin films improves as a result of ion implantation.

The shape of the diffraction peak is influenced by structural effects, such as crystallite size and strain caused by lattice defects [28]. To quantify the contributions of crystallite size and strain, the Williamson–Hall (W-H) method was used. The Williamson–Hall equation is given as [19].

(4) $$\frac{\beta cos\theta }{\lambda }=\frac{1}{D^{2}}+\frac{4\varepsilon sin\theta }{\lambda }.$$

Here, λ is the X-ray wavelength (1.54060 Å), θ is the Bragg angle, D is the crystallite size in nm, ε is the strain, and β is the FWHM in radians.

**Figure 9:**
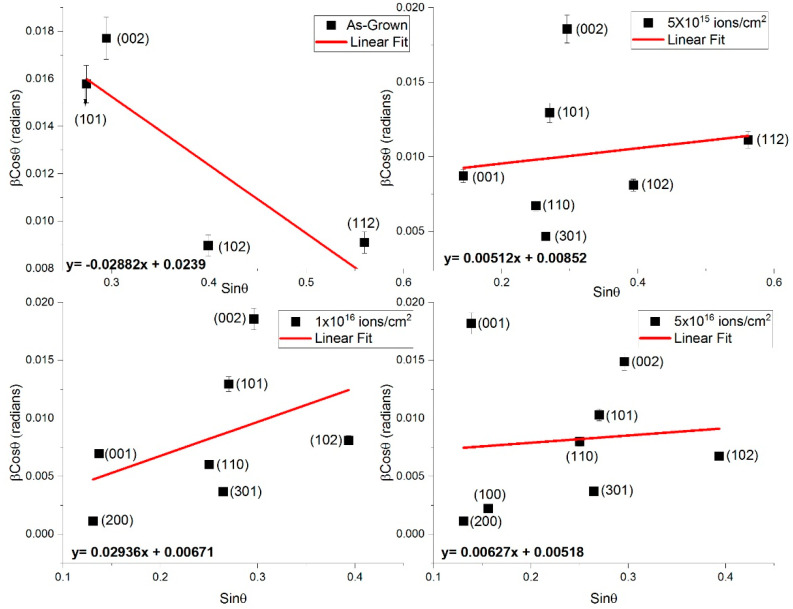
Williamson–Hall analysis for the deconvoluting size and strain broadening by considering the peak broadening of reflection as a function of 2θ of the as-grown and V^+^ ion-implanted ZnO thin films at fluences of 5 × 10^15^, 1 × 10^16^, and 5 × 10^16^ ions/cm^2^.

Using Equation (4) and the diffraction peaks corresponding to the (101), (002), (102), and (112) planes (for as-grown) and (200), (001), (101), (110), and (301) planes (for V^+^ ion-implanted ZnO), the contributions of crystallite size and strain to peak broadening were determined. Figure 9 shows the linear fitting of the W-H plots for as-grown and ion-implanted ZnO thin films at different fluences.

**Figure 10:**
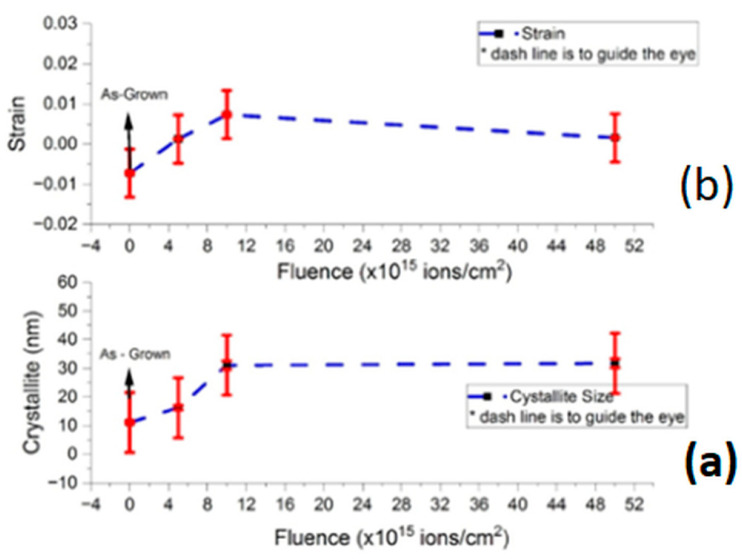
Crystallite size (**a**) and strain (**b**) of V^2^ ion-implanted ZnO thin films in comparison with as-grown using the Williamson–Hall method as a function of ion fluence.

From Figure 10, crystallite size and strain significantly influence peak broadening. The W-H plots exhibit a negative slope for as-grown films, indicating minimal strain contribution to broadening. After ion implantation, the slope becomes positive, suggesting the increasing contribution of strain as ion fluence increases.

The average crystallite sizes, determined from the inverse intercepts of the W-H plots, are recorded in Table 4, Column 3, and illustrated in Figure 10 as a function of ion fluence. The slopes of the W-H plots represent the average strain. Crystallite size increases exponentially, reaching an upper limit of approximately 26.17 nm at a fluence of 5 × 10^16^ ions/cm^2^. This trend is consistent with the Scherrer method, as shown in Figure 8 and Figure 10 and Table 5, Column 4.

The observed increase in grain size can be attributed to strain relaxation, defect annealing during the implantation process, and the possible formation of new phases induced by ion implantation. These factors contribute to the formation of larger grains, as confirmed quantitatively by the XRD patterns.

The variation in strain with ion fluence, as determined using the W-H method, is presented in Figure 10. The strain increases progressively with ion fluence, driven by several physical mechanisms during V^+^ ion implantation, as suggested by the TRIM simulation results (Section 3.1). These mechanisms include:

1. Defect Formation:

V^+^ ion implantation introduces various defects into the ZnO crystal lattice, including vacancies and interstitials (point defects, Figure 4b and Figure 6 and dislocations (extended defects). As ion fluence increases, the defect density grows, resulting in localized strain due to variations in atomic spacing and cumulative lattice distortion.

2. Stress Relaxation:

With the accumulation of defects, the ZnO lattice attempts to relax the induced stresses. However, this relaxation is often incomplete, leading to a net increase in strain as the material balances the competing forces of defect-induced distortion and stress redistribution.

3. Structural Modifications:

Ion implantation can cause phase transitions or changes in the grain structure of ZnO. These structural modifications further contribute to the increase in strain by altering the material’s inherent lattice parameters and introducing additional mismatches.

#### 3.2.3. Interplanar Spacing

As-grown and V^+^ ion-implanted ZnO thin films on borosilicate glass substrates consist of atoms separated by distances, *d*, which are resolved into multiple planes and recorded in Table 5, Column 3, as interplanar spacings ($d_{hkl}$*—*spacings). Each spacing corresponds to a unique $d_{hkl}$*—*spacings value. To investigate the effect of ion fluence on these spacings, the average $d_{hkl}$*—*spacings values between *d_hkl_* planes of atoms were determined for as-grown and V^+^ ion-implanted thin films at fluences of 5 × 10^15^,1 × 10^16^ and 5 × 10^16^ ions/cm^2^. These values were found to be 0.0294, 0.0240, 0.0192, and 0.0183 Å, respectively, along with the orientation of a single crystal.

**Figure 11:**
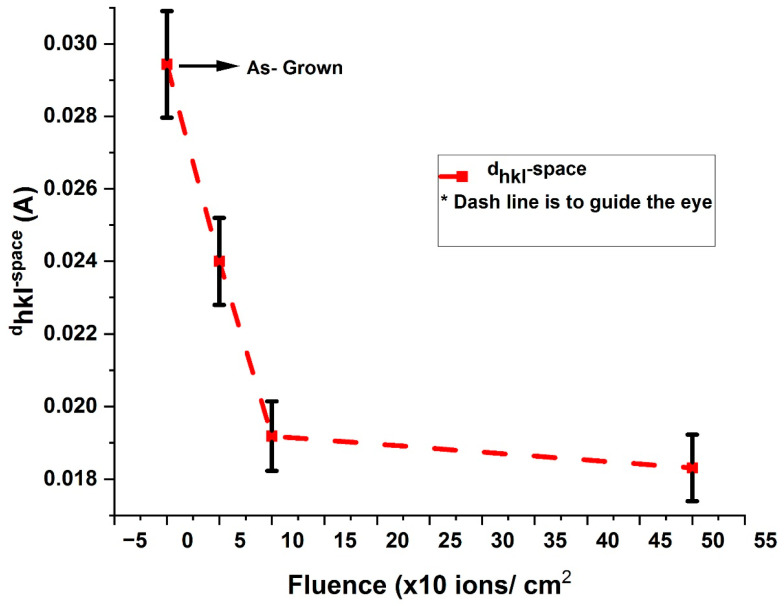
Variation in $d_{hkl}$-spacings in as-grown and V^+^ ion-implanted ZnO thin films as a function of fluence.

Figure 11 illustrates the effect of ion fluence on the average $d_{hkl}$*—*spacings between the planes of atoms in the V^+^ ion-implanted ZnO thin films compared to the as-grown films. From Figure 11, it can be observed that the $d_{hkl}$*—*spacings value decreases progressively, showing a reduction of 37.76% from 5 × 10^15^ to 5 × 10^16^ ions/cm^2^ compared to the as-grown films. This decrease in $d_{hkl}$*—*spacings after ion implantation is attributed to grain boundary relaxation, an increase in the density of the V^+^ ion-implanted ZnO thin films, and the phase transformation induced by the implantation process.

#### 3.2.4. Lattice Constants

The crystal structure of ZnO is described as a hexagonal close-packed (hcp) structure with two distinct phase angles: $a=b=90^{{}^{\circ}}$ and $c=120^{{}^{\circ}}$ [23]. Consequently, the lattice constants a and *c* were calculated using the following relations [23].

(5) $$a=\sqrt[{d_{hkl}}]{\left(h^{2}+k^{2}+l^{2}\right)};\;\mathrm{and}$$

(6) $$\frac{1}{d^{2}}=\frac{4}{3}\;\left(\frac{h^{2}+hk+k^{2}}{a^{2}}\right)+\frac{l^{2}}{c^{2}},$$

where hkl are the Miller indices, a and *c* are the lattice constants, and d is defined in Section 3.2.3.

**Figure 12:**
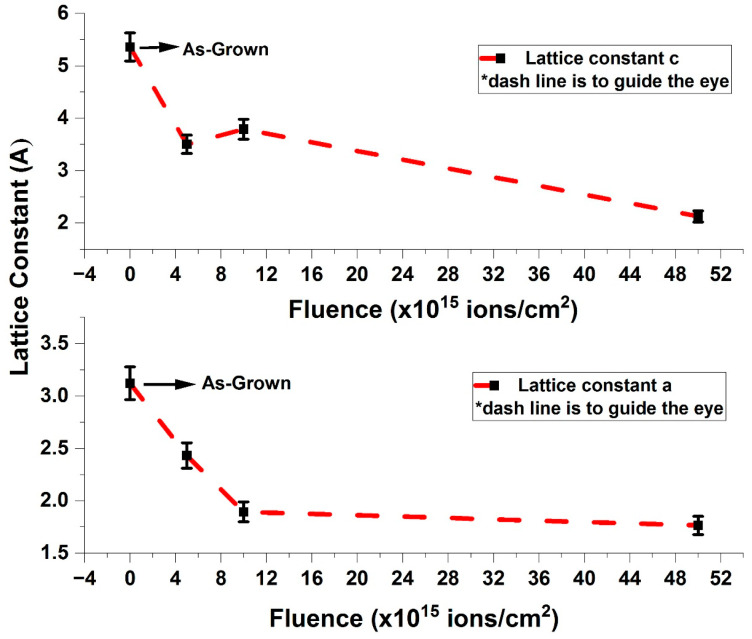
Variation in lattice constants c and a in as-grown and V^+^ ion-implanted ZnO thin films as a function of ion fluence.

Using Equations (5) and (6), the variations and estimated lattice constants a and *c* are presented in Figure 12 and recorded in Table 5, Columns 5 and 6. From Figure 12, the average values of a and *c* are 3.1219 ± 0.1271 Å and 5.3587 ± 0.1527 Å, respectively.

It can be observed that as ion fluence increases, there is a gradual decrease in the lattice constants up to a fluence of 1 × 10^16^ ions/cm^2^ compared to the as-grown sample. Beyond this, the decrease is slight, reaching minimum values of 1.7656 Å and 2.1278 Å for lattice constants a and *c*, respectively, at 5 × 10^16^ ions/cm^2^ (Figure 12). This suggests that the lattice constants decrease with increasing ion fluence. A similar trend was observed in Co-doped ITO films by Stankiewicz et al. [29].

#### 3.2.5. Dislocation Density

**Figure 13:**
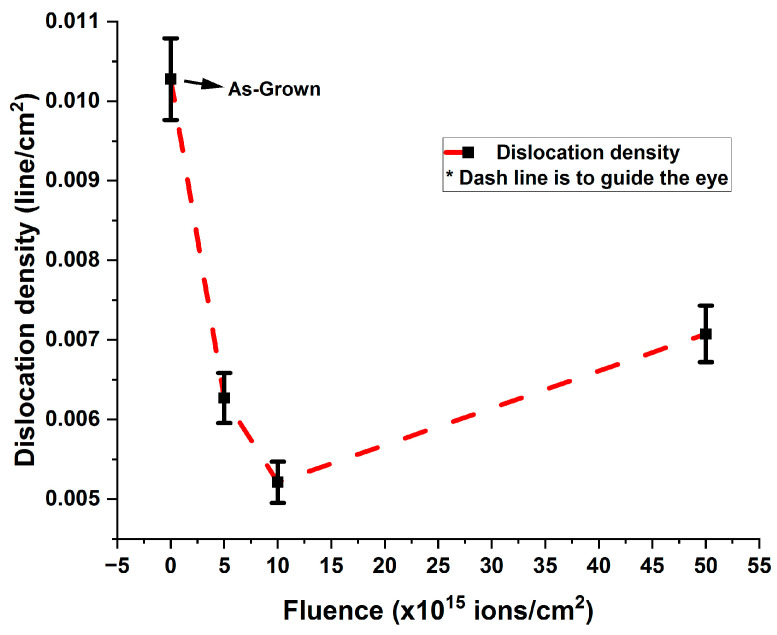
Variation in dislocation densities of as-grown and V^+^ ion-implanted ZnO thin films as a function of ion fluence.

Dislocations represent lattice misregistrations, leading to imperfections in the crystal matrix. The dislocation densities were calculated using Equation (7) [26]

(7) $$\;\delta =\frac{1}{D^{2}},$$

where D is defined in Equation (3). The estimated dislocation densities for as-grown and V^+^ ion-implanted ZnO thin films on borosilicate glass substrates are recorded in Table 6, Column 2, and illustrated in Figure 13.

From Figure 13, it is evident that there is a rapid decrease in dislocation density with increasing ion fluence up to 1 × 10^16^ ions/cm^2^ compared to the as-grown sample. Beyond this fluence, there is a slight increase up to 5 × 10^16^ ions/cm^2^. This observation can be attributed to grain boundary relaxation and annihilation in the V^+^ ion-implanted ZnO thin films, indicating improved stability.

#### 3.2.6. Morphological Properties

**Figure 14:**
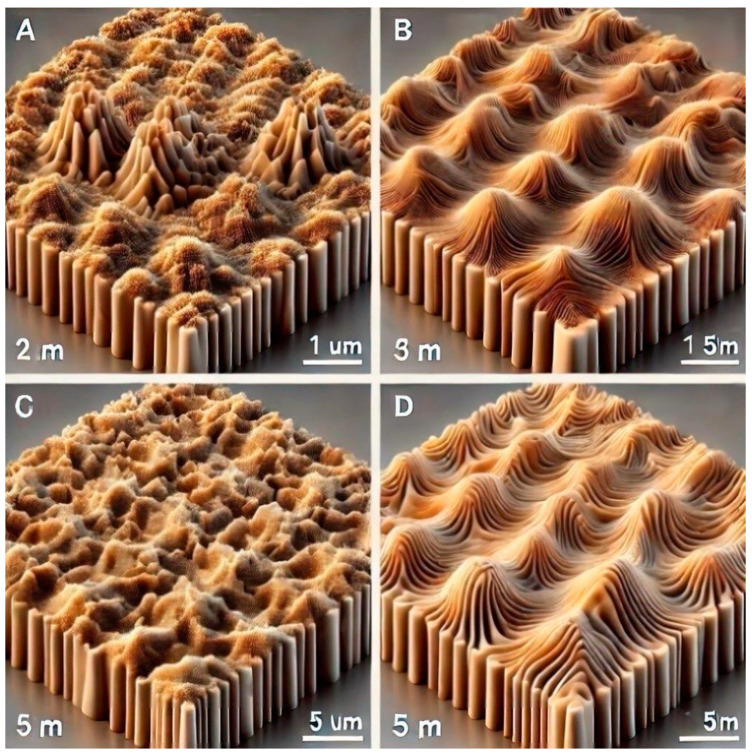
AFM 3D images of (**A**) as-grown and V^+^ ion-implanted ZnO thin films at fluences of (**B**) 5 × 10^15^, (**C**) 1 × 10^16^, and (**D**) 5 × 10^16^ ions/cm^2^.

**Figure 15:**
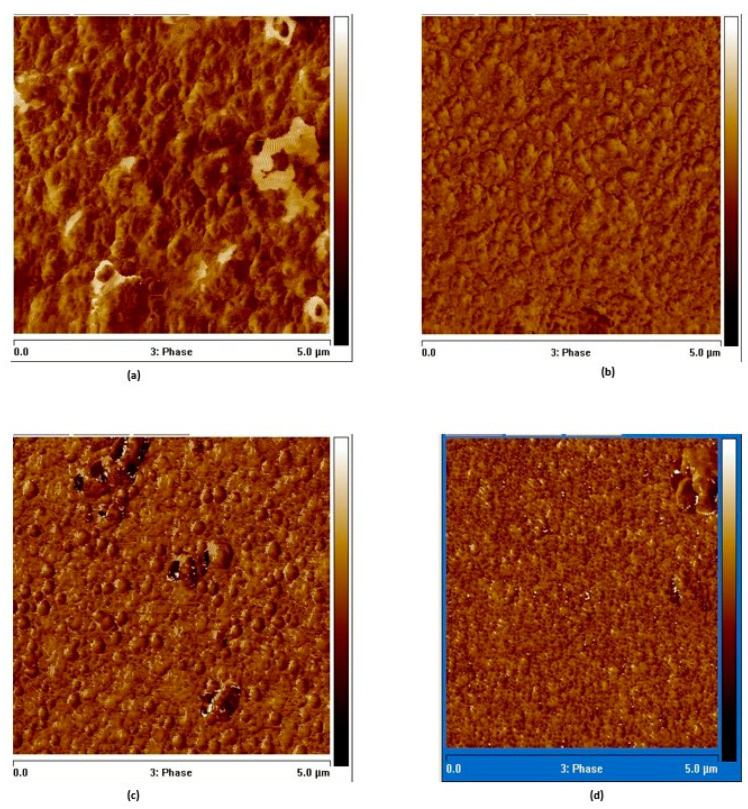
AFM 2D images of (**a**) as-grown and V^+^ ion-implanted ZnO thin films at fluences of (**b**) 5 × 10^15^, (**c**) 1 × 10^16^, and (**d**) 5 × 10^16^ ions/cm^2^.

**Figure 16:**
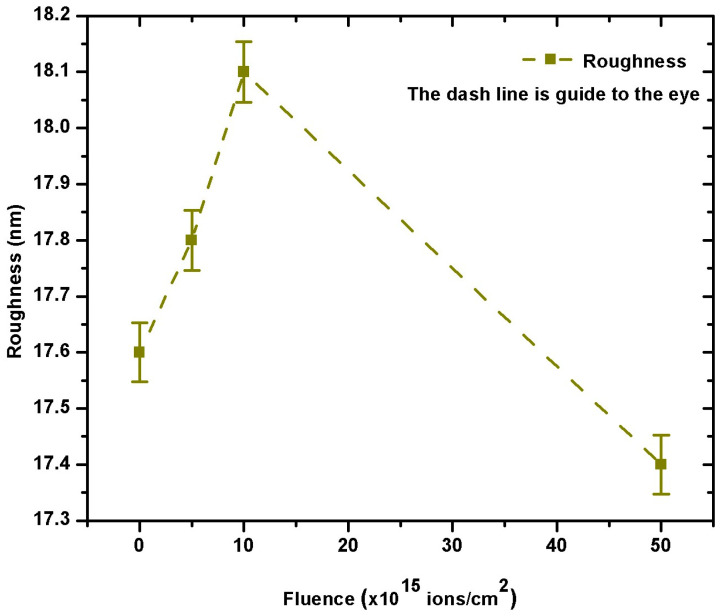
Surface roughness as a function of fluence for as-grown and V^+^ ion-implanted ZnO thin films.

Figure 14 and Figure 15 depict 3D and 2D AFM scans of as-grown and V^+^ ion-implanted ZnO thin films at different ion fluences. The calculated root mean square (RMS) and average roughness values are listed in Table 7 and Figure 16, it is evident that the surfaces of thin films implanted with fluences ranging from 5 × 10^15^ to 5 × 10^16^ ions/cm^2^ were modified upon implantation. The AFM image of the thin film implanted at 5 × 10^16^ ions/cm^2^ shows a sharp decrease in roughness, with an RMS roughness of approximately 17.4 nm and a grain size of 31.8162 nm. For comparison, the as-grown thin film had an RMS roughness of 17.6 nm and a grain size of 11.1826 nm.

Moreover, the increase in grain size resulted in a more regular distribution of granular-shaped crystallites (Figure 7). The decrease in RMS roughness also established a relatively smooth surface [30]. However, the roughness of the thin film implanted at 1 × 10^16^ ions/cm^2^ increased by 2.84%.

The observed changes in surface roughness and grain size with increasing ion fluence are driven by a combination of physical and chemical effects. Thus, the following key mechanisms and impact were considered to provide an in-depth explanation of the mechanisms behind these changes.

1(a) Mechanism: V^+^ ions deposit energy into the ZnO thin films, increasing local temperatures and enhancing atomic mobility on the surface

1(b). Impact: Enhanced surface diffusion can smooth out certain features while creating new ones, modifying both surface roughness and grain morphology.

2(a). Mechanism: The energy from the V^+^ ions causes atoms in the ZnO thin films to mix and redistribute. This mixing resulted in localized changes in density or surface topology.

2(b). Impact: The redistribution of material contributes to variations in surface roughness and can blur or redefine grain boundaries.

3(a). Mechanism: The V^+^ ion species implanted at 170 keV determine the penetration depth and energy transfer to the ZnO thin films.

3(b). Impact: The implantation parameters influence the extent of sputtering, defect generation, and grain evolution, thereby affecting the observed surface changes.

In comparison with recent studies, this roughness is lower than that reported for Yb and Eu oxide-doped ZnO films and aligns with the trend observed in Nb-doped ZnO films, suggesting that ion implantation at this dose effectively smooths the film surface [6,31].

### 3.3. Thermal and Chemical Stability

Using the EXPO2014 software [18], isotropic refinements were performed with the average factors calculated from simulated crystal structures to determine thermal parameters. The B-factors, which indicate the relative vibrational motion of different parts of the crystal structure due to thermal effects, are also known as Debye–Waller factors. For a given atom in a stationary position, its atomic scattering factors, f, change to $f_{T}$, as follows [32,33]:

(8) $$f_{T}=fexp(-B{sin}^{2}\theta /\lambda^{2}),$$

where *T* represents temperature, and B is mathematically defined as $B=8\pi^{2}<\pi^{2}>,<\pi^{2}>=U$ representing the mean square vibrational amplitude. This theoretical calculation was employed to estimate the thermal and chemical stabilities of ZnO thin films.

**Figure 17:**
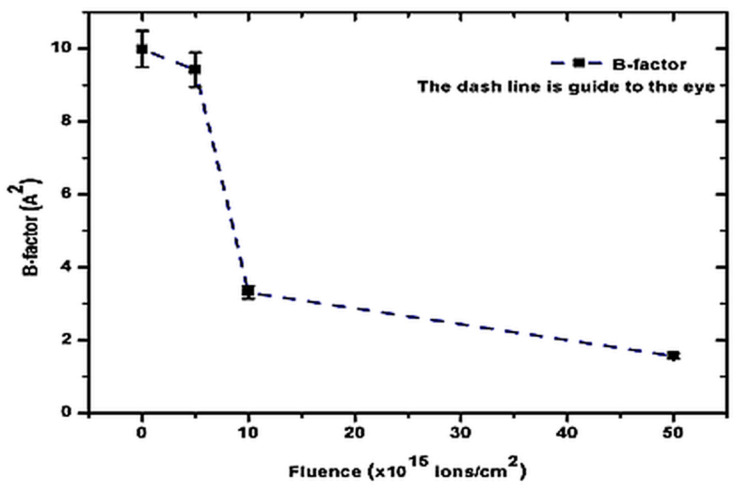
Variation in thermal factors (B-factors) of as-grown and V^+^ ion-implanted ZnO thin films as a function of ion fluence.

By implementing Equation (8) in EXPO2014, the thermal factors (B-factors) and mean square displacement amplitude (U) of as-grown and V^+^ ion-implanted ZnO thin films were determined. The results are recorded in Table 8 and shown in Figure 17.

From Figure 17, it can be seen that the vibrational motion of different parts of the V^+^ ion-implanted ZnO thin film structures, induced by thermal motion, decreases rapidly from a fluence of 5 × 10^15^ to 5 × 10^16^ ions/cm^2^. This indicates that atoms in the thin film implanted at 5 × 10^16^ ions/cm^2^ are stable due to the low B-factor of 15.70%. However, atoms in the thin film implanted at a fluence of 5 × 10^15^ ions/cm^2^ are flexible due to the high B-factor of 94.29%. Additionally, the mean square displacement amplitude was observed to decrease with increasing fluence.

The observed thermal stability trends align well with studies on other ion-implanted thin films. Higher fluences induce densification and defect stabilization, reducing atomic mobility and vibrational amplitudes, while lower fluences leave the material more thermally flexible due to isolated defects [33,34].

### 3.4. Chemical Bond Analysis

Fourier transform infrared spectroscopy (FTIR) is a tool which is widely used to probe the vibrational properties of materials. FTIR relies on the absorption of photons by the material’s molecules, where the absorption peaks and band positions depend on the material’s morphology, structure, and chemical composition [35,36].

**Figure 18:**
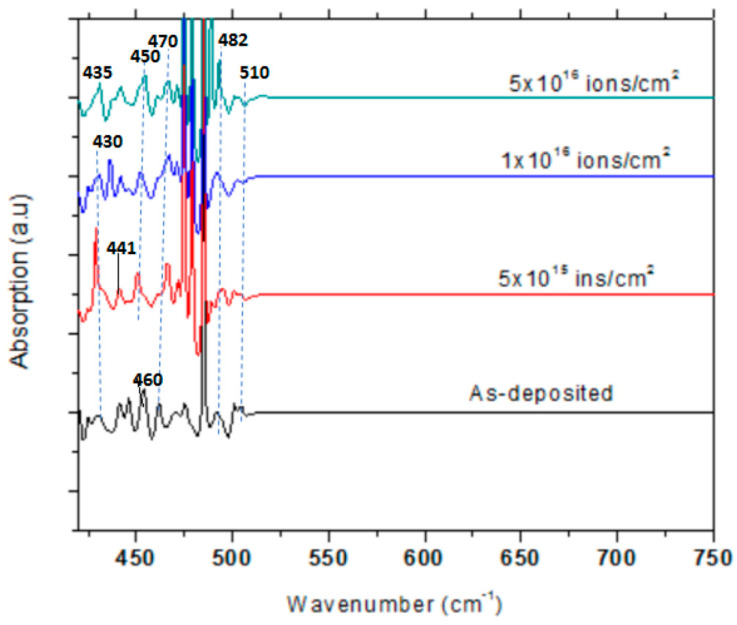
FTIR spectra in the wavenumber range 420–750 cm^−1^ at room temperature of as-grown and V^+^ ion-implanted ZnO thin films at fluences of 5 × 10^15^, 1 × 10^16,^ and 5 × 10^16^ ions/cm^2^.

Figure 18 shows the compositional analysis of as-grown and V^+^ ion-implanted ZnO thin films at different fluences (5 × 10^15^, 1 × 10^16^, 5 × 10^16^ ions/cm^2^), performed using FTIR at 300 K in the range of 420–750 cm^−1^.

The presence of ZnO thin-film stretching vibrations is confirmed by peaks in the range of 470–482 cm^−1^ for as-grown films and 435–482 cm^−1^ for V^+^ ion-implanted films [37] The absence of vanadium oxide peaks in the FTIR spectrum implies the effective implantation of vanadium ions into the ZnO lattice rather than phase separation due to their similar ionic radii, as discussed earlier (Section 1), and the introduction of lattice strain [37]. This incorporation has modified the ZnO lattice dynamics and influenced its functional properties, such as optical bandgap and defect states, Section 3.5 and Figure 4b, respectively. The absence of vanadium oxide and the incorporation of vanadium into the ZnO lattice correlate highly with shifts in diffraction peaks, particularly the (002) plane of ZnO (Section 3.2.1).

Figure 18 also reveals that most peaks in the as-grown films appear as doublets, which change to single peaks in the V^+^ ion-implanted ZnO thin films. An absorption shoulder at 430 cm^−1^, which is present in as-grown films, is absent at higher fluences. The absorption peak shifts to a higher wavenumber (435 cm^−1^) for films implanted at 5 × 10^16^ ions/cm^2^, corresponding to the polycrystalline phase of the films. Additionally, an absorption shoulder at 510 cm^−1^ shifts to a lower wavenumber (500 cm^−1^).

These observations suggest that microstructural changes lead to the well-resolved peaks at 430, 435, 441, and 450 cm^−1^ and the shifts to 435 and 500 cm^−1^ in the V^+^ ion-implanted films. The absorption shoulders and strong peaks in the range of 475–479 cm^−1^ are observed in both as-grown and V^+^ ion-implanted ZnO thin films.

### 3.5. Optical Properties

The optical transmittance (T) and absorption of the as-grown and V^+^ ion-implanted ZnO thin films on borosilicate glass substrates were analyzed using UV–Vis spectroscopy and the Tauc model [21]. The Tauc model defines the relationship between the absorption coefficient (α) and the incoming photon energy (hν) as:

(9) $$({\alpha h\nu )}^{2}=C(h\nu -E_{g}),$$

where C is a constant, $E_{g}$ is the optical band gap, and the power of two indicates a direct transition in ZnO. The absorption coefficient (α) is given by:

α=2.303A/t, where A is the absorbance and t is the film thickness.

**Figure 19:**
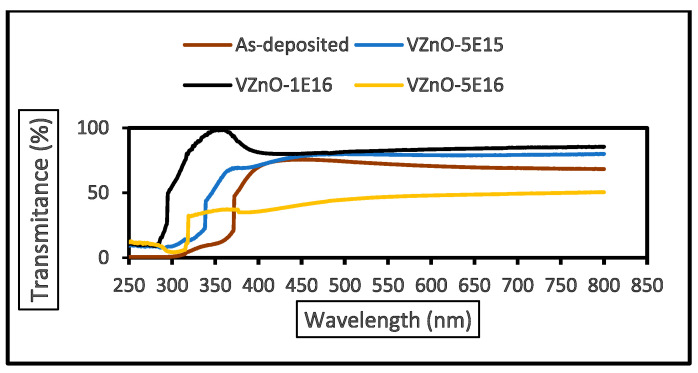
Optical transmittance of as-grown and V^+^ ion-implanted ZnO thin films at different fluences.

**Figure 20:**
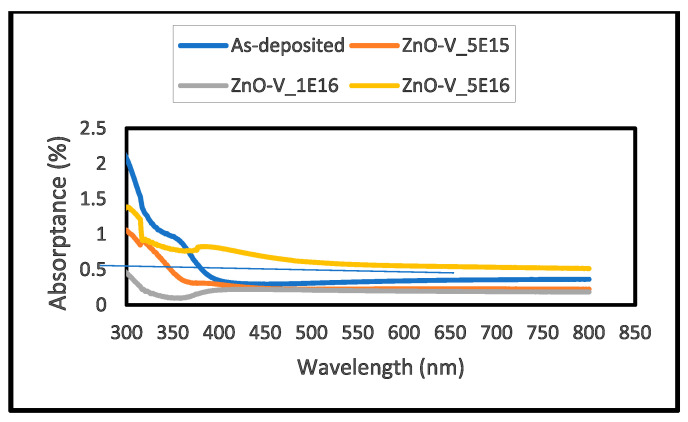
Optical absorbance of as-grown and V^+^ ion-implanted ZnO thin films at different fluences.

**Figure 21:**
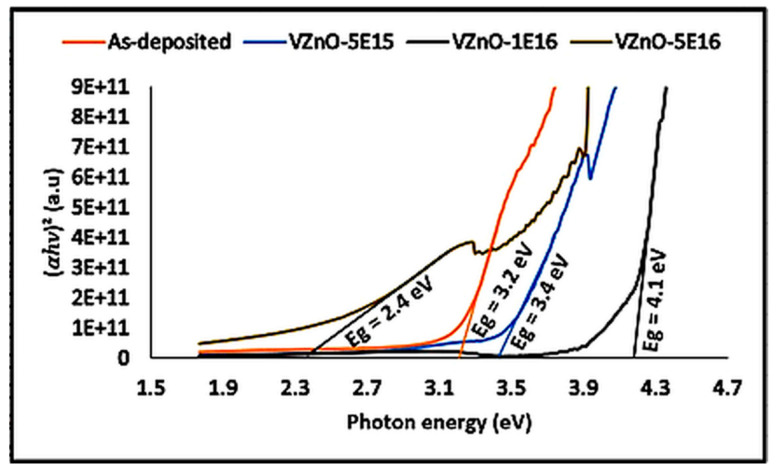
Calculated optical band gap, Eg, of as-grown and V^+^ ion-implanted ZnO thin films at different fluences using Tauc relation.

Figure 19, Figure 20 and Figure 21 illustrate the UV–Vis spectral properties of as-grown and V^+^ ion-implanted ZnO thin films at fluences of 5 × 10^15^, 1 × 10^16^ and 5 × 10^16^ ions/cm^2^. The estimated optical band gap energies ($E_{g}$) are recorded in Table 7. As shown in Figure 21, the optical band gap energies vary between 2.4 and 4.1 eV. Significant changes in the band gap energies are observed due to ion implantation. An increase in bandgap energy is observed with fluence up to 1 × 10^16^ ions/cm^2^, which is most likely due to a combination of density of state engineering and structural changes. The correlation between structural changes (strain, crystallite size) and optical bandgap is that increased lattice strain and observed crystallite size, from 5 × 10^15^ to 1 × 10^16^ ions/cm, enhance the bandgap due to quantum confinement and strain-induced band modifications. However, strain relaxation, observed crystallite size and defect accumulation at a fluence of 5 × 10^16^ ions/cm^2^, lowering the bandgap by introducing defect states. This bandgap narrowing further shifts the absorption edge to longer wavelengths.

The observations of bandgap variations with ion fluence are consistent with previous findings [28,38]. For instance, the increase in bandgap energy with Mg-doped ZnO thin films is consistent with the initial bandgap widening observed in this work at ion fluence up to 1 × 10^16^ ions/cm^2^ [28].

Figure 19 and Figure 20 depict the transmittance and absorbance behaviors, respectively, for as-grown and ion-implanted films. The transmittance progressively increases from as-grown to 1 × 10^16^ ions/cm^2^, with the highest transmittance of 82.34%. At this fluence, the lowest absorbance of 0.12% is observed, with the absorption edge shifting to a lower wavelength (368 nm), indicating a blue shift. However, recent findings by Nur-E-Alam et al. (2025) [38] in “Tailoring Ga-doped ZnO Thin Film Properties: Argon Flow Rate Modulation and Dynamic Sputtering Geometry Analysis”, indicate that optimizing argon flow rates and dynamic sputtering geometry can significantly enhance the optical and electrical properties of Ga-doped ZnO films. Thus, the high transmittance in Ga-doped ZnO films compared well with transmittance levels observed at lower fluences in this study [8,38].

Conversely, the thin film implanted at 5 × 10^16^ ions/cm^2^ exhibits the highest absorbance (0.56%) and lowest transmittance (45.26%). The significant drop in transmittance is caused by increased light scattering from surface defects and absorption by defect states within the bandgap [38].

In the ultraviolet range (280–380 nm), transmittance shoulders observed in ion-implanted films (5 × 10^15^ to 5 × 10^16^ ions/cm^2^) are likely due to substrate reflection [28].

## 4. Conclusions

The study demonstrated that V^+^ ion implantation significantly influences the structural, optical, chemical bond, and thermal properties of ZnO thin films deposited by rf-magnetron sputtering. Ion implantation effectively modified the morphology, crystallite size, strain, dislocation density, and lattice constants, leading to enhanced optical properties such as increased bandgap and transmittance, alongside reduced absorbance. These improvements were especially pronounced at a fluence of 1 × 10^16^ ions/cm^2^, which exhibited an optimal balance between enhanced optical performance and structural stability. Furthermore, the findings showed that thermal factors decreased with higher ion fluences, contributing to the improved stability of the implanted films.

The results affirm the potential of V^+^ ion implantation as a method to tailor ZnO thin films for specific applications in optoelectronic devices, such as solar cells and LEDs, as well as energy nanodevices. This modification of ZnO thin films might enable devices to achieve optimum efficiency performance in real-world applications compared to unmodified films. Future research should focus on further optimizing implantation parameters to achieve desired property enhancements while maintaining long-term stability in diverse operating environments.

## Figures and Tables

**Table 1:** SRIM-calculated implantation energy, thin film thickness, and ion fluence, together with projected range, Rp, in ZnO thin films on borosilicate glass substrates.

Ion Fluence (×10^15^ ions/cm^2^)	Energy (keV)	Film Thickness (nm)	Ion Range, Rp, (nm)
5	170	120	73.98
10	170	120	73.98
50	170	120	73.98

**Table 2:** TRIM-2013 calculated parameters for V^+^ ion-implanted ZnO on borosilicate glass substrates at 170 keV.

Fluence (×10^15^ ions/cm^2^)	Dopant Conc. (×10^20^ atom/cm^3^)	Vacancy Conc. (×10^23^ vac./cm^3^)	Critical Energy Dens. (×10^17^ eV/cm^3^)	dpa (×10^00^)
5	5.65	6.8	6.95	8.19
10	11.3	13.6	13.9	16.4
50	56.5	68	69.5	81.9

**Table 3:** TRIM-2013 calculated displacement energy, electronic and nuclear energy loss for V^+^ ion-implanted ZnO thin films.

Displacement Energy (keV/ion)	Nuclear Energy Loss (keV/nm)	Electronic Energy Loss (keV/nm)
6.66	9.28	7.06

**Table 4:** The intensity ratio, 100(I/I max), W-H average size, and strain of V^+^ ion-implanted ZnO thin films in comparison with as-grown.

Fluence (×10^15^ ions/cm^2^)	Intensity Ratio (×10^−2^ a.u)	W-H (nm)	Strain, (×10^−4^)
0 (As-grown)	23.92	5.80	−72.05
5	26.21	16.28	12.80
10	35.21	20.67	73.40
50	47.90	26.77	15.68

**Table 5:** Structural parameters of V^+^ ion-implanted ZnO thin films in comparison with as-grown.

Fluence (×10^15^ ions/cm^2^)	2θ (deg)	*d_hklspace_* (×10^−2^ Å)	FWHM (×10^−1^ deg)	*h k l*
0 (As-grown)	31.83	2.1124	9.7238	101
	34.27	2.2699	11.0075	002
	47.08	3.0774	5.9354	102
	68.09	4.31367	6.8136	112
5	16.54	1.1080	5.0449	001
	28.98	1.9281	3.9742	110
	30.69	2.0390	2.7639	301
	31.37	2.0833	7.7066	101
	34.45	2.2814	11.134	002
	46.34	3.0318	5.0449	102
	68.38	4.3294	7.7067	112
10	15.03	1.0083	0.7053	200
	15.78	1.0573	4.3176	001
	28.98	1.9281	3.9742	110
	30.69	2.0390	2.7639	301
	31.37	2.0833	7.7066	101
	34.45	2.2813	11.134	002
	46.34	3.0317	5.0449	102
50	15.04	1.0083	0.7053	200
	15.96	1.0696	11.2714	001
	17.98	1.2040	1.4652	101
	28.98	1.9281	5.9777	110
	30.69	2.0390	2.7639	301
	31.37	2.0833	7.7066	101
	34.45	2.2814	11.134	002
	46.34	3.0317	5.0449	102

**Table 6:** Structural parameters such as strain, dislocation density, crystallite size, and lattice constant of as-grown and V^+^ ion-implanted ZnO thin films at different ion fluences.

Fluence (×10^15^ ions/cm^2^)	Dislocation Density, δ (×10^−12^ line/m^2^)	Crystallite Size (nm)	Lattice Constant, a (Å)	Lattice Constant, c (Å)
0 (As-grown)	13.8484	8.4977	1.4937	2.1124
	17.5232	7.5543	2.2699	4.5397
	4.6891	14.6034	3.4407	6.1548
	5.0478	14.0750	5.2831	8.6276
Average	10.2771	11.1826	3.1219	5.3587
5	3.9473	15.9167	0.554	1.1080
	2.3447	20.6519	1.3634	0.0000
	1.1251	29.8134	2.724	2.0390
	8.7181	10.7010	1.4731	2.0833
	17.9110	7.4721	2.2812	4.5628
	3.4066	17.1333	3.3896	6.0635
	6.4361	12.4648	5.2525	8.6592
Average	6.2698	16.31	2.43226	3.5023
10	7.7430 × 10^−2^	113.6436	1.0081	0.0000
	2.8966	18.5803	0.5287	1.0573
	2.3447	20.6519	1.3634	0.0000
	1.1251	29.8134	3.2215	2.0390
	8.7181	10.7010	1.4726	2.0833
	17.9110	7.4721	2.2813	4.5628
	3.4066	17.1332	3.3896	6.0635
Average	5.2114	31.1435	1.8950	3.7910
50	7.7430 × 10^−2^	113.6436	1.0079	0.0000
	19.7320	7.1189	0.5348	1.0696
	3.3168 × 10^−1^	54.9084	0.8512	1.204
	5.3045	13.7302	1.3634	0.0000
	1.1251	29.8134	3.224	2.039
	8.7181	10.7010	1.4731	2.0833
	17.9110	7.4721	2.2814	4.5628
	3.4066	17.1332	3.3896	6.0635
Average	7.0758	31.8162	1.7656	2.1278

**Table 7:** Optical band gaps, transmittance, average roughness (Ra) at different ion fluences, together with root mean squares (RMS), wavelengths, and corresponding absorbance edges of V^+^ ion-implanted ZnO thin films.

Fluence (×10^15^ ions/cm^2^)	Wavelength (nm)	Absorbance (au)	Bandgap (eV)	Transmittance (%)	RMS (Rq) (nm)	Ave. Ra (nm)
0	415	0.31	3.20	72.14	17.6	12.6
5	456	0.23	3.40	78.50	17.8	12.6
10	368	0.12	4.10	82.34	18.1	9.35
50	589	0.56	2.40	45.26	17.4	9.07

**Table 8:** Isotropic (iso) refined thermal parameters (B and U) of as-grown and V^+^ ion-implanted ZnO thin films at different ion fluences.

Fluence (×10^15^ ions/cm^2^)	B-Factor *A*^2^	U (×10^−1^ *A*^2^)
0	9.985	1.9784
5	9.415	1.1924
10	3.308	0.4189
50	1.568	0.1986

## Data Availability

All data were included in the manuscript.
